# Editorial: Misunderstanding Others: Theory of Mind in Psychological Disorders

**DOI:** 10.3389/fpsyg.2022.838853

**Published:** 2022-02-07

**Authors:** Manuel Sprung, Juliane Burghardt, Monica Mazza, Friedrich Riffer

**Affiliations:** ^1^Department of Psychology and Psychodynamics, Division of Clinical Psychology, Karl Landsteiner University of Health Sciences, Krems an der Donau, Austria; ^2^Waldviertel Psychosomatic Center, Eggenburg, Austria; ^3^Department of Applied Clinical Sciences and Biotechnology, University of L'Aquila, L'Aquila, Italy

**Keywords:** theory of mind, social cognition, metacognition, mentalizing, borderline personality disorder, autism spectrum disorder, schizophrenia, maternal depression

Theory of mind (ToM) or mentalizing is a fundamental component of human cognition. It is defined as the ability to ascribe mental states, such as desires, beliefs, intentions, and emotions, to oneself and others and to explain and predict behavior based on these mental states (Wimmer and Perner, 1983). ToM is also a part of current models of empathy, which include two ToM systems, a cognitive and an affective system, with separate but interacting brain networks (Shamay-Tsoory, 2011). The cognitive ToM system involves thinking about thoughts, intentions, or beliefs. The affective ToM involves thinking about feelings and is distinct from the affective empathy system, which involves sharing the emotional experiences of others (Shamay-Tsoory, 2011). Important developmental ToM milestones are in preschool, school-age, and early adolescence (Rakoczy, 2017; Meinhardt-Injac et al., 2020; Wellman, 2020; Devine and Lecce, 2021; Osterhaus and Koerber, 2021). Childhood adversity can disrupt ToM development (Benarous et al., 2015). There are also limits in healthy adults' ToM use (Apperly et al., 2010); ToM abilities typically declines in aging adults (Henry et al., 2013). ToM is also linked to social development and functioning. For instance, ToM is related to prosocial behavior, peer popularity and reciprocated friendship (Fink et al., 2015; Slaughter et al., 2015; Imuta et al., 2016). Moreover, superior ToM relates to less loneliness, less social rejection, and being rated as socially skilled by teachers (Banerjee et al., 2011; Devine et al., 2016; Koerber and Osterhaus, 2020).

In the last two decades ToM impairments have also been reported in over 30 different mental disorders, including autism spectrum disorder (ASD), schizophrenia, borderline personality disorder (BPD), post-traumatic stress disorder, depression, and eating disorders. Dysfunctional ToM has been proposed as a transdiagnostic clinical marker (Cotter et al., 2018) and has been included as a subconstruct in the Research Domain Criteria, a research framework for investigating mental disorders (Cuthbert, 2014). The underlying mechanisms and the precise role of ToM in the etiology, diagnosis, and treatment of these mental disorders are also still poorly understood. Some existing treatment programs, such as mentalization-based therapy or metacognitive training, emphasize ToM treatment as a core component (Moritz et al., 2014; Malda-Castillo et al., 2019). This Research Topic promotes our understanding on the specific nature of ToM in individuals with borderline personality disorder, autism spectrum disorder and schizophrenia.

The study by Lévay et al. investigated whether individuals with BPD exhibit an asymmetry between their own social behavior and their expectations of other people's social motivation. Compared with health control subjects, individuals with BPD expected significantly more selfishness in others. This finding suggests that ToM impairments in BPD are best characterized as mistrust and a negativity bias. This specific profile of ToM impairment may be linked to an abusive family environment, common in the history of many BPD patients, in which cooperation was met with selfishness.

A different profile of impaired ToM, indicated by difficulties in the spontaneous intuitive ascription of mental states, has been observed in individuals with ASD. Krämer et al. investigated the feasibility and efficacy of a treatment program specifically targeting spontaneous ToM in individuals with ASD, an adapted Mentalization-Based Treatment (MBT). The ToM abilities of a group of adult patients with Asperger's syndrome, a form of ASD with no general impairments in language or cognitive abilities, significantly improved over the course of the treatment. Despite the lack of a control group this is a very promising finding, showing that MBT can help AS patients to better understand and reflect on interpersonal situations.

Two further papers in this Research Topic address other aspects related to ToM, in patients with psychosis and schizophrenia. Lysaker et al. provide an interesting review of research on social cognition, intersubjectivity and metacognition in individuals with psychosis. This article points out that deficits in social cognition cannot fully explain the disturbed social functioning of individuals with psychosis. Instead Lysaker et al. suggest that deficits in metacognitive capacity, contributing to experiences of self and others as increasingly fragmented, provide a better account of social dysfunction in psychosis. Maaßen et al. address emotional awareness an important aspect of affective ToM. They examining the usability and convergent validity of the Levels of Emotional Awareness Scale (LEAS) in a large sample of outpatients with schizophrenia or schizoaffective disorders. The LEAS uses scenarios of social situations to evoke participants' emotions and assess both their own emotional reactions and their inferences about the emotions of interactions partners presented in the scenarios. Their findings suggest that although formal levels of EA may not differ significantly between patients and healthy controls there may be inadequacies in other-related mentalizing in the patient group.

Finally, Nonnemacher et al. offer a developmental perspective on ToM. In an impressive longitudinal design, this study investigated the relation of self-regulatory skills in infancy with later ToM abilities in pre-school age children of clinically depressed mothers and healthy controls. Contrary to the authors hypothesis maternal depression did not impair ToM developmental *per se*. However, an interaction was found between infants self-comforting behavior and later ToM abilities. This finding adds to the heterogeneous picture about the influence of parental mental illness on children's ToM development, suggesting that other factors such as self-regulatory abilities contribute to ToM development.

## Author Contributions

MS wrote the first draft. JB reviewed and revised the manuscript. MM and FR reviewed the manuscript. All authors contributed to the article and approved the submitted version.

## Funding

The Gesellschaft für Forschungsförderung Niederösterreich (GFF) [Society for Research Promotion Lower Austria] supported this work by financing the endowed professorship in clinical psychology held by MS. The VAMED Institute for Gender Medicine financed the position held by JB.

## Conflict of Interest

The authors declare that the research was conducted in the absence of any commercial or financial relationships that could be construed as a potential conflict of interest.

## Publisher's Note

All claims expressed in this article are solely those of the authors and do not necessarily represent those of their affiliated organizations, or those of the publisher, the editors and the reviewers. Any product that may be evaluated in this article, or claim that may be made by its manufacturer, is not guaranteed or endorsed by the publisher.
